# Predictive modeling of optimism bias using gray matter cortical thickness

**DOI:** 10.1038/s41598-022-26550-y

**Published:** 2023-01-06

**Authors:** Raviteja Kotikalapudi, Dominik A. Moser, Mihai Dricu, Tamas Spisak, Tatjana Aue

**Affiliations:** 1 Institute of Psychology, University of Bern, Fabrikstrasse 8, 3012 Bern, Switzerland; 2 Department of Neurology, University Hospital Essen, Hufelandstrasse 55, 45147 Essen, Germany

**Keywords:** Cognitive neuroscience, Computational neuroscience, Social behaviour, Social neuroscience

## Abstract

People have been shown to be optimistically biased when their future outcome expectancies are assessed. In fact, we display optimism bias (OB) toward our own success when compared to a rival individual’s (personal OB [POB]). Similarly, success expectancies for social groups we like reliably exceed those we mention for a rival group (social OB [SOB]). Recent findings suggest the existence of neural underpinnings for OB. Mostly using structural/functional MRI, these findings rely on voxel-based mass-univariate analyses. While these results remain associative in nature, an open question abides whether MRI information can accurately *predict* OB. In this study, we hence used predictive modelling to forecast the two OBs. The biases were quantified using a validated soccer paradigm, where personal (*self* versus *rival*) and social (*in-group* versus *out-group*) forms of OB were extracted at the participant level. Later, using gray matter cortical thickness, we predicted POB and SOB via machine-learning. Our model explained 17% variance (R^2^ = 0.17) in individual variability for POB (but not SOB). Key predictors involved the rostral-caudal anterior cingulate cortex, pars orbitalis and entorhinal cortex—areas that have been associated with OB before. We need such predictive models on a larger scale, to help us better understand positive psychology and individual well-being.

## Introduction

Optimism bias (OB) is a well-established behavioral dimension, where individuals associate themselves with more positive than negative outcome scenarios^1,2^. More specifically, OB can further be branched into the deeper concepts of personal and social OBs^3^. Personal OB (POB) stems from a bias towards oneself where an individual expects a more favorable outcome compared to a peer or competitor, given the same scenario. For example, ‘I have better chances to be accepted for this job position than the fellow competitor’. On similar grounds—yet different—social OB (SOB) is manifested in comparing successful outcomes for one’s own (or preferred) social group when compared to their rival social group. An example could be, ‘my favorite soccer team will likely win at tonight’s match’. Both, POB and SOB can be quantified as the difference in the likelihood estimated for a positive outcome for *self (in-group) versus rival (out-group)*, where positive estimates index the presence of OB. The presence or absence of such a bias is quantified by psychological questionnaires, for example the comparative optimism scale^1^, as well as through experimental paradigms^4–7^.

Psychological evaluations through behavioral questionnaires and experimental results may validly and reliably reveal OB. For a more comprehensive picture, studies have also investigated the neural correlates of OB, predominantly using fMRI. For example, studies have reported an active role of the anterior cingulate cortex, ACC^8–10^ and ventromedial prefrontal cortex, vmPFC^9,11^. Other important areas include the orbitofrontal cortex (OFC), posterior cingulate gyrus (PCC), striatum, and insula^4,9,11^. But very few studies have directly probed into the neuroanatomical association of OB or related constructs via structural MRI using T1-weighted images. For example, participants in a recent study^12^ performed an expectancy task that addressed optimism robustness, which is expressed by greater attentional guidance by optimistic rather than pessimistic cues. Said study found that gray matter volume (GMV) of the ACC, bilateral insular, and primary visual cortex, all correlated with optimism robustness^12^. Using a related concept of belief update (preferential updating of future expectancies for pieces of information that support an optimistic rather than pessimistic view of the own future), a study has reported, in older recruits only, that GMV of the ACC is linked with OB^13^. But this phenomenon was not observed in the same study, in the younger population. While these studies thoroughly investigated the role of brain structure and function in concepts underlying OB, it is still unclear as to what is the predictive yield of these structures towards OB.

Studying such predictive yield is worthwhile because it may enable the identification of the most important structural brain characteristics (e.g., of identified key regions in earlier research, such as the ACC, insula, striatum, and PCC) to the strength of the manifested OB. Lai et al. have addressed this issue to some extent in their study, where bilateral GMV of the putamen correlated with trait optimism (i.e., the tendency to look positively into one’s own future, which is not necessarily irrational as in OB)^14^. However, their primary findings were based on voxel-based morphometry with behavioral questionnaires of the life orientation test (LOT, as an indicator for trait optimism), and its results were only then confirmed by a predictive approach, as a post-hoc reliability strategy. Hence, unsupervised predictive modeling was not the primary choice of analysis. Yet, such studies always remain as an important step towards robust predictive modeling using dedicated machine-learning approaches. Importantly, by forming a predictive model for OB, we can enhance these frameworks to better explain the structure-driven psychological state of well-being, as OB and related concepts have been shown to be closely linked with individual well-being^15,16^. Not only that, but a predictive marker for OB can also find potential applications in understanding neural substrates of depression, post-treatment recovery and acute-chronic pain states, all of which have been strongly linked to OB and similar concepts^15,17–21^. For example, OB plays a crucial role in treatment expectations^22^. To elaborate it further, inducing optimism bias significantly improves health outcomes when patients are given positive information about their treatment or illness along with support and reassurance (in contrast to formal consultation or consultations with no reassurance at all). By quantifying OB, clinicians can have greater insights into treatment expectations for better health outcomes. Such an approach may thus inspire deeper clinical and psychological applications.

**Figure 1:**
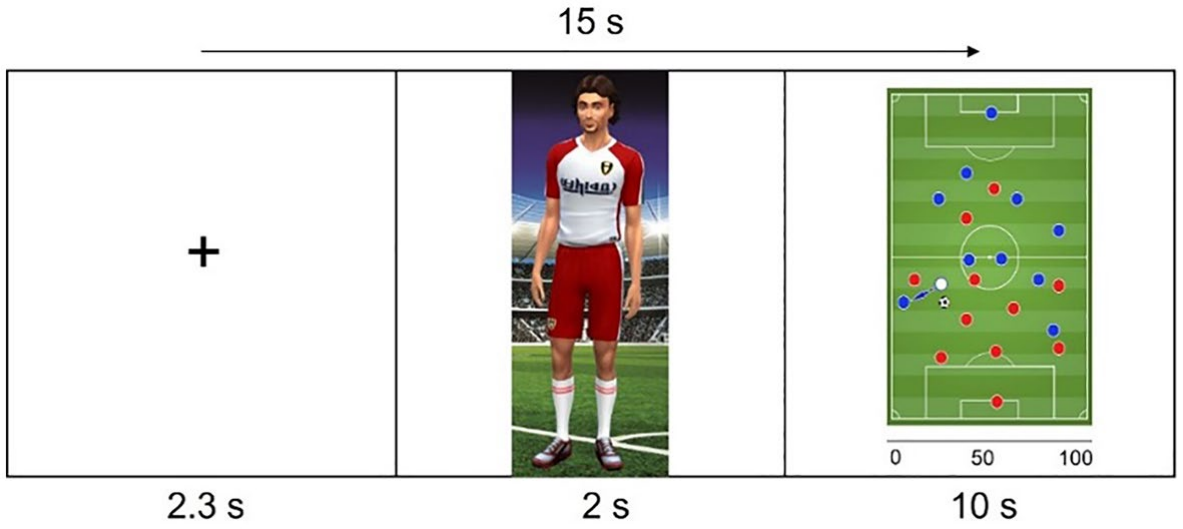
Soccer task. At the start of each trial, a fixation cross was presented for a duration of 2–3 s (variation due to jittering). Next, a player representing the self, rival, in-group, or out-group was displayed for 2 s. Subsequently, a soccer game scenario was presented for 10 s; in this latter phase participants estimated the likelihood for a successful pass (rating range 0–100%). Each trial lasted approximately 15 s.

In our study, we aimed at deriving such a predictive model using well established and reliable measures of gray matter cortical thickness, exclusively utilizing a machine-learning framework. Gray matter cortical thickness remains the feature set for the predictive modeling, and POB and SOB serve as the target variables. Both, POB and SOB were calculated from a previously established soccer-based experimental paradigm^3,4,23^. In brief, each participant recruited for our experiment was asked to estimate the likelihood for successful outcomes given different soccer situations (Fig. 1). Most importantly, the participant would give these likelihood estimates for the self, a personal rival, an in-group (a favorite team) and an out-group (rival team). POB was quantified as the difference in estimates for self-versus rival, and SOB was quantified as the difference in estimates for in-group versus out-group. The neuroanatomical parameters were trained using a linear model in a machine-learning framework, to predict both POB and SOB. Overall, our approach was aimed at finding the true predictive value of gray matter cortical thickness in explaining OB. We examined both POB and SOB to identify potential overlapping brain-structural similarities and divergences between the two.

## Results

### Behavioral results

We performed outlier detection on the target variables (i.e., POB and SOB) and excluded one participant, given that their scores for both POB and SOB deviated more than 3SD from the sample mean. This resulted in a total of 45/46 participants qualifying for the final analysis. The excluded participant gave comparatively lower likelihood estimates for both the rival (rivalscore = 37.25, sample mean rival score = 52.53), and the out-group (out-group score = 35.63, sample mean out-group-score = 53.88) in comparison to estimates for self and in-group (self score = 59.54, sample mean self score = 55.10; in-group score = 54.88, sample mean in-group score = 56.05).

**Figure 2:**
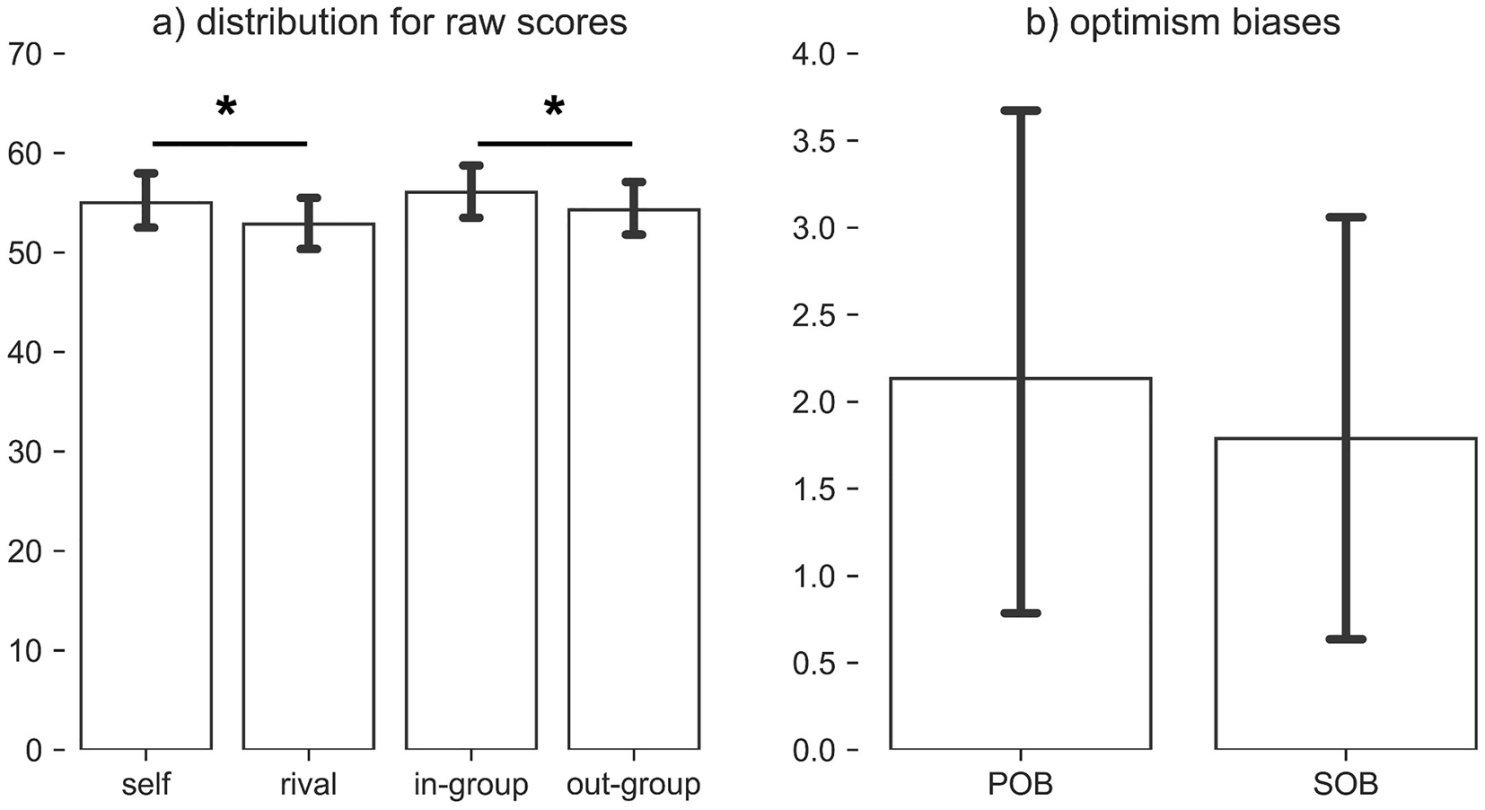
Behavioral data. (**a**) Behavioral data for the raw scores (self, rival, in-group, and out-group) and (**b**) the derived measures (POB, SOB) are presented. * Indicates a significant difference in the means (*p* < 0.05). Derived measures are POB = self—rival, and SOB = in-group—out-group, where POB stands for personal optimism bias and SOB for social optimism bias. Both POB and SOB are referred to as targets in the machine learning framework. Error bars represent standard errors.

The behavioral data are displayed in Fig. 2. To check our hypothesis that POB (= self > rival) and SOB (= in-group > out-group) manifests, we performed a paired t-test for both the contrasts. Both POB (t (44) = 2.97, *p* = 0.002) and SOB (t (44) = 2.96, *p* = 0.002) were significant. There were no significant differences between POB versus SOB (t (44) = 0.462, *p* = 0.646).

### Machine-learning results

We derived a predictive model for estimating individual variability in OBs, i.e., on the personal and social domains of optimism. For the predictive modelling, the gray matter cortical thickness measures (34 regions per hemisphere) served as the features and POB and SOB, as the target measures (measures that the model estimates). We used a linear regression model with least absolute shrinkage and selection operator—LASSO, for the purpose of model fitting and predictions. Our analyses framework was supported by a nested cross-validation (CV) approach to prevent information leakage/overfitting between the train and test data folds. Finally, the predictions were further checked for any confounding effects from age, sex and estimated total intracranial volume (TIV)—to obtain robust leakage-free and confounder-free estimates of POB and SOB.

**Figure 3:**
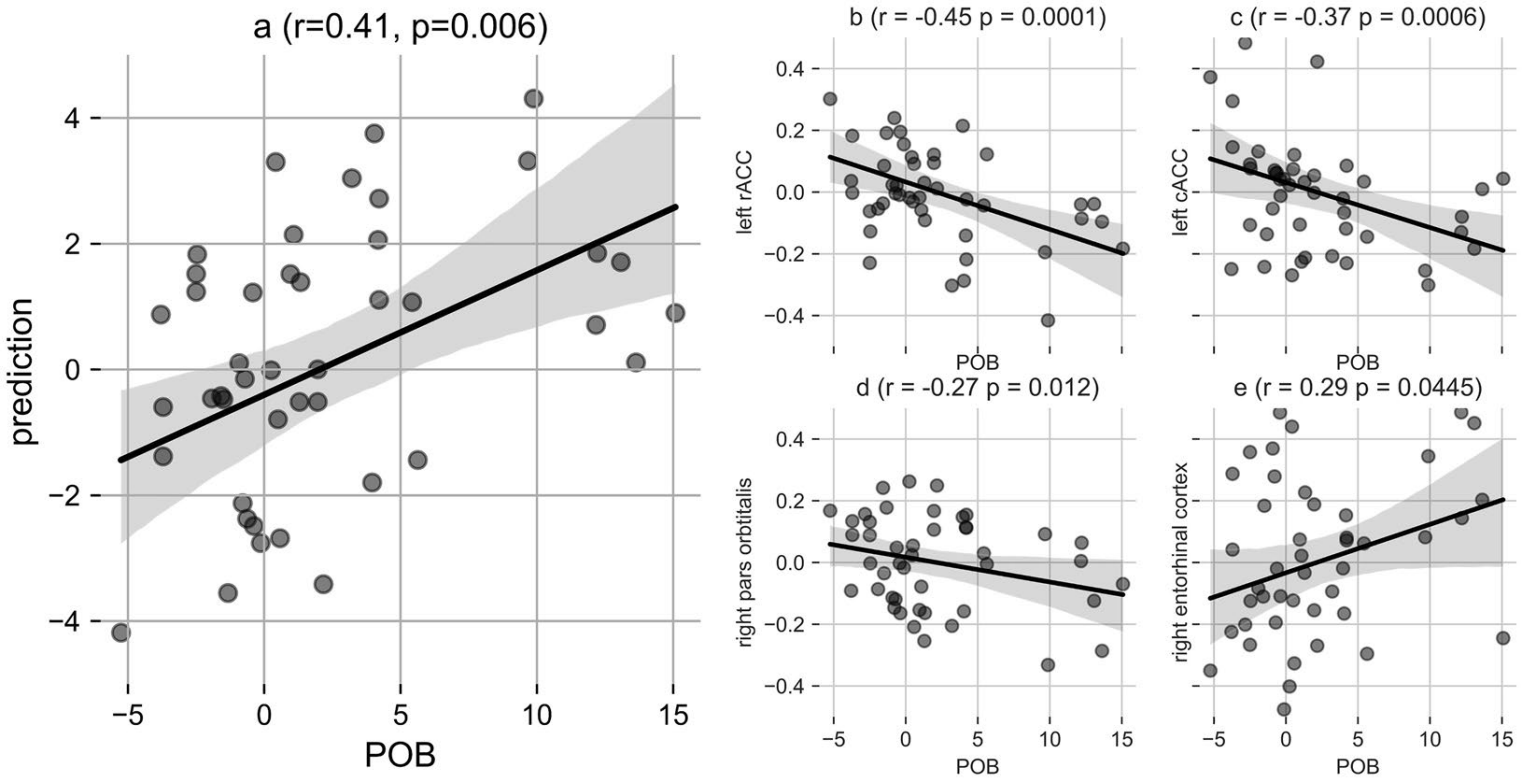
Predictions for personal optimism bias (POB). (**a**) The scatter plot with regression line is presented for prediction versus actual POB scores, (**b**–**e**) indicate predictive regions. For **b**–**e**, age was regressed out from each of the regions. 10,000 surrogate combinations were tested to generate the *p*-value (*p*) for the Pearson’s r.

For POB predictions, the best LASSO model was found to be with regularization factor α = 0.1. The unbiased estimates (predictions) correlated with the target with Pearson’s r = 0.41, *p* = 0.006. The variance explained by our model amounted to 17%, i.e., R^2^ = 0.17. The prediction versus target and predictors and their significant weights are presented with Fig. 3 and Table 1, respectively. Significant negative weighted regions (cortical thickness of predictive regions that correlated significantly with POB at *p* < 0.05) were the left rostral anterior cingulate (rACC), left caudal anterior cingulate (cACC) and right pars orbitalis. The significant positive weighted region was the right entorhinal cortex. The final unbiased predictions (following regressing out their effects) did not correlate significantly anymore with age, sex or TIV, thus eliminating addressing any potential confounding effects (Table 2).

**Table 1:** Mean weights for nested cross-validation best estimator (unbiased model).

Regions	Weights
Left rostral anterior cingulate (rACC)	− 10.59
Left caudal anterior cingulate (cACC)	− 3.84
Right pars orbitalis	− 0.30
left entorhinal	− 0.05
Left isthmus cingulate	0.00
Left pericalcarine	0.01
Right frontal pole	0.02
Right postcentral	0.55
Right entorhinal	1.75
Left parahippocampal	2.73

‘−/ + ’ sign indicates a negative/positive correlation of the corresponding region with personal optimism bias (POB).

**Table 2:** Predictions with and without confounding effects.

	Prediction versus POB (r, *p*)	Predictions versus age (r, *p*)	versus sex (r, *p*)	versus eTIV (r, *p*)
Before conf. effects corr	(0.45, 0.003)	(0.34, 0.02)	(0.03, 0.8)	(0.06, 0.7)
After conf. effects corr	(0.41, 0.006)	(− 0.01, 0.93)	(− 0.04, 0.8)	(0.01, 0.95)

It can be observed that *predictions with confounding effects* are confounded by ‘age’. Hence, the potential confounder effects were regressed out from the final predictions, in the nested cross-validation framework, to obtain unbiased estimates of personal optimism bias (POB). (r, *p*) refers to Pearson’s r, and *p*-value tested for 10, 000 permutations. eTIV = estimated total intracranial volume. conf. = confounding, corr. = correction.

## Discussion

In this paper, we describe a predictive model for OB in a machine-learning framework, using gray matter cortical thickness and experimentally-derived scores of POB and SOB. The model significantly predicts POB with Pearson’s r = 0.41 (*p* = 0.006, permutations = 10, 000). The Pearson’s r is in the small to medium range in terms of its effect size. The key predictors include the left rostral anterior cingulate cortex (rACC), left caudal anterior cingulate cortex (cACC), right pars orbitalis and right entorhinal cortex. The predictions were exclusively driven by cortical thickness and not confounded by the measures of age, sex, and total intracranial volume (TIV). Our predictive model hence reveals important brain structural associations with OB and is a step towards (a) predictive modeling of such biases. Moreover, the current findings may (b) inspire clinical applications for mental disorders like depression and (c) increase our understanding of psychological well-being.

### Model considerations

First, we have used the derived measures of POB and SOB as target variables in our predictive modeling, thus, two different kinds of OB to compare potential brain-morphological similarities between them. Second, the paradigm implemented to derive these biases has been validated previously^3,4,23^. Third, as a feature set, we considered gray-matter cortical thickness for predicting optimism bias. One strong reason favoring such a selection is that cortical thickness is a robust and reliable measure widely used in neuroimaging studies, and requires minimal feature scaling and residualization (e.g. cortical thickness measures are not driven by TIV), thereby facilitating a simple and reliable interpretation of the findings^24,25^. Fourth, confounding effects play a crucial role in predictive modeling, as the model can learn from the confounders. For example, while the residualization methods vary across studies, removing confounder effects from the initial feature set as a standard protocol do not guarantee a confounder-free prediction due to the inadequacy of such residualization methods^26^. Hence, we regressed out the confounding variables from the final predictions (unbiased estimates), thereby providing a clear bifurcation of predictions into (a) predictions with confounder effects and (b) predictions free from confounding effects. Here, it is worth mentioning that the regression of confounders was performed within the nested CV to avoid data leakage. Fifth, regular linear regression models can be prone to overfitting e.g., in including the final number of features for predicting the dependent variable. This issue can be resolved to an appreciable extent by using the LASSO model^27^, which applies a shrinkage to irrelevant variables (by assigning a zero weight through regularization, i.e. by adding a penalty), thereby reducing overfitting and, at the same time, increasing the interpretability of the findings. Hence, our selection of the linear model as LASSO was predetermined. Furthermore, using the non-nested CV approach, we kept the training and test datasets separate for model selection and hyperparameter tuning, thereby avoiding any cross-contamination (minimal data leakage) and avoiding ‘optimistic’ predictions by the model^28^. This is because the data point on which prediction is performed is not used in selecting the best model estimator (i.e., model used to make the estimations/predictions).

### Biological relevance

The strongest predictors of our POB model, after regressing out the confounding effects, were the right rostral and caudal anterior cingulate cortex (ACC). This was followed by the right pars orbitalis (orbital part of inferior frontal gyrus, IFG) and right entorhinal cortex. In terms of effect sizes, both parts of the ACC (rostral + caudal) were in the medium range. The ACC is reported to play a crucial role in influencing OB and related concepts, as per significant findings from both functional^8–10,29^ and structural studies^3,12,30^. For instance, in a concept close to OB, GMV of the ACC was shown to correlate negatively with optimism robustness^12^. In this study, optimism robustness was expressed by greater attentiveness towards reward expectation (i.e., optimistic) cues and less towards punishment expectation (i.e., pessimistic) cues. While the task is different to the soccer paradigm in the current study, both address related facets of personal optimism and the (negative) directionality of the link between ACC size and optimism is comparable. Furthermore, in another study, a reduced ACC volume was associated with quality of life, which was acquired with a self-report questionnaire from participants^31^, a concept that is positively associated with optimism^32–34^. Both structural and functional studies have indicated an active involvement of the ACC in fear and punishment^35,36^, which fits well with the notion that a decreased volume of ACC could be indicative of lower self-referential fear or punishment upon failing. Such observations are consistent with our current finding of higher estimates for one’s own successful outcomes against a rival's. It thus is possible that a small-sized ACC promotes POB (please note that causality still needs to be proven).

Another region, the right inferior frontal gyrus (orbital part of IFG) was also a negative predictor of POB. This observation provides support to Sharot et al.^37^ finding for the right IFG, which was negatively correlated to optimistic belief updating. In their experiment on OB based on belief updates, BOLD signal in the right IFG was negatively correlated with undesirable error estimates. That is, maintenance of personal optimism went along with decreased brain activity in the right IFG when the informed average probability for a negative event to occur was higher than a participant’s estimate and indicated the rejection of learning based on negative feedback. In our study also, we found a negative association between IFG thickness and POB, demonstrating that not only functional but also structural aspects of this area play an important role in positive expectancy biases.

The only significant positive correlation with POB in the current study was found for the right entorhinal cortex, a region that surrounds the hippocampus as part of the hippocampal formation^38,39^. It is known for communicating neocortical inputs to the hippocampus^40,41^, and is well established for its contributions to memory encoding^42–45^. A positive correlation for this region’s thickness with POB might explain a selective encoding and consideration for more positive than negative perspectives, with such regulative actions preventing depression^46^.

Finally, remaining left (parahippocampal, entorhinal, isthmus cingulate, pericalcarine) and right (frontal pole, postcentral) hemispheric regions were also assigned non-zero weights by the best estimator LASSO in the POB model. However, individually, these regions did not correlate significantly with POB. Hence, these regions—alone—may not have the predictive capacities towards the target, but together with other regions, they help in predicting POB.

We did not find any regions supporting SOB. The main reason for this could be that the model simply did not retain any of the regional thickness measures as significant, and most importantly as generalizable predictors for SOB. While saying so, other structural imaging metrics such as white matter connectivity could prove to be better estimators for such a bias, because SOB is evident from the behavioral aspect (soccer paradigm) and may be explained by a different set of structural markers. Hence, while we see similarities between POB and SOB in some studies^3,23,47^, the current data outline that there are also clear discrepancies. Here, it is further worth mentioning that previously, using the Stereotype Content Model^48,49^, Moser et al.^47^ were able to identify cortical thickness associates for social optimism. However, this investigation differed in various aspects to the current study, including experimental paradigm (e.g., inclusion of various out-groups and positive as well as negative scenarios in the case of Moser et al.) and type of multivariate analysis implementation in use (sparse canonical correlation analysis versus machine-learning based model selection).

### Limitations

One limitation of our study is the sample size (n = 45) and testing the model for external datasets. We resolve this issue of false positives and model overfitting to an extent through our analysis framework. That is, (a) incorporating a nested cross-validation approach (the nested cross-validation approach produces unbiased estimates of target variables for lower and higher sample size datasets^50^), (b) intentionally using a LASSO regression that accommodates dimensionality reduction to address for multicollinearity—thereby—reducing the number of features given the sample size (number of features versus number of samples), (c) restricting higher model parameters by avoiding external feature selection and feature scaling methods that can lead to multiple modelling approach-based hypotheses testing, and (d) using reliable target variables that are derived from an established experimental paradigm (i.e., soccer game based quantification of OB^3,4,23,51^ (in contrast to self-reported questionnaires)). Incorporating such robust modelling steps for brain-wide association can prove to be effective even when training models with much lower sample sizes (n = 20–40)^52,53^. Hence, we expect the model to be generalizable towards newer datasets, though the findings shall be interpreted with caution. Second, to ensure the stability and reliability of our model, we chose not to investigate the second- and third-order interactions for target predictions. While interactions may have provided additional insights, they would have increased the feature set and, given the lower sample size, could have made the model unstable. By not including the interaction, we were able to maintain a stable model. Nevertheless, in the future, multi-collaborative research consortiums could help in addressing these issues to some extent. 

## Conclusion

We derived predictive models to predict OB using gray-matter cortical thickness as the feature set, and personal-social levels of optimism (POB and SOB) as the dependent variables. The model predictions significantly correlated with POB, and the predictions were not confounded by factors such as age, sex and TIV. The major predictors for POB were thickness in the left rostral and caudal anterior cingulate gyrus, right pars orbitalis and right entorhinal cortex, predominantly correlating negatively with POB. While the predictive framework was able to estimate POB, a similar result was not achieved in predicting SOB. Our predictive model approach for POB is a step towards predictive neuroimaging in positive psychology, which could find potential applications in explaining states such as individual well-being, as well as in identifying clinically-relevant symptoms for conditions such as acute and chronic depression.

## Methods

### Participants

Our study takes leverage from Moser et al.^3^, who investigated functional brain correlates of OBs. We have used the same dataset in terms of participants, experimental setup, and deriving the OBs. But, while Moser et al. investigated the functional correlates for OBs, we use the structural MRI images in a machine-learning framework for predictive modeling of OBs. In brief, we recruited 46 participants (34 females) at the University of Bern, Switzerland through flyers, emails, and the local participant pool. The recruited participants’ age group was in the range of 19–35 years (mean = 22.9, std = 3.62). Exclusion criteria for the recruitment were self-reported neurological disorders, psychoactive substance abuse and left-handedness. At the data processing level, we excluded participants with either of the behavioral biases (POB or SOB) with 3 standard deviations away from the sample mean. The local ethics committee of the University of Bern approved all experiments, methods of data collection, data handling and analyses. Informed consent was obtained from all participants in accordance with the guidelines of the Declaration of Helsinki. Participants were paid either 25 CHF/hour or obtained course credits. All methods were performed in accordance with the relevant guidelines and regulations.

### Experimental setup

The experimental participation took place inside an MRI scanner (3T Magnetom, Prisma from Siemens, Erlangen, Germany) and the task was designed using e-prime 2.0 Professional (Psychology Software Tools, Sharpsburg, PA, USA). The task cartoons were created with The Sims 4 (Electronic Arts, Redwood City, California, USA) and the soccer scenarios with Photoshop CS6 (Adobe Inc., San Jose, California, USA). Visual projection of the task was enabled by an LCF projector (PT-L711E, Panasonic, Kadoma, Japan). The task’s duration was approximately 30 min. Moser et al.^3^ gives a detailed description of the experimental paradigm, which has been adapted from a similar experiment on American football^4^. Participants were trained on this experiment before the actual task was conducted. In brief, the participants were shown four different characters who faced 16 identical soccer scenarios. The four characters refer to (a) the self (where participants consider themselves to be confronted with a given scenario), (b) a personal rival (participants consider a personal competitor for their own position in their soccer club undergoing that same scenario), (c) an in-group (consideration of a player from a team the participants identify with), and (d) an out-group (consideration of a player from the in-group’s rival team). Given these characters and scenarios, the participants’ task was to estimate the likelihood for a successful pass (i.e., passing the ball to a fellow team player), or a successful goal. The likelihood estimates were given by button presses that shifted a slider on an analogue scale that ranged from 0 (certain of an unsuccessful outcome) to 100 (certain of a successful outcome). The task was performed in two blocks (a + b and c + d) with randomized trials (16 scenarios for each character) within each block. Using the likelihood estimates, four raw scores (for self, rival, in-group, and out-group) and two derived scores of OB were calculated (POB = self—rival, where positive value indicates the presence of a POB; SOB = in-group—out-group, where a positive value indicates the presence of a SOB).

### Magnetic resonance imaging (MRI) scanning protocol:

MRI measurements were conducted with a 3T scanner (MAGNETOM Prisma, Siemens, Erlangen—Germany) using a 64-channel head coil at the University Hospitals Bern, Switzerland. We used an MPRAGE sequence (magnetization-prepared rapid gradient-echo) with TR (repetition time) = 2300 milli seconds (ms), TE (echo time) = 2.98 ms, and TI (inversion time) = 900 ms, flip angle = 9°, matrix size = 160 × 256 × 256, and with an isotropic spatial resolution of 1 mm^3^.

### Features and targets

At first, the T1-weighted DICOM images were converted to NifTI format using a conversion software called dcm2niix (https://github.com/rordenlab/dcm2niix/releases, accessed on 19th of November 2022). The T1-weighted anatomical scans were independently processed using freesurfer version 6.0 (https://freesurfer.net/,^54,55^) to obtain gray-matter cortical thickness measures. The surface-based morphometry approach of freesurfer has been validated and detailed extensively elsewhere^54,56–58^. In brief, the native space T1-weighted image of the participant was registered to a standard space using a Talairach transformation^59^, and white matter (WM) labelling was performed considering their location and intensity, followed by intensity normalization^55,60^. Subsequent processes included skull stripping^61^, hemispheric separation (based on locations of corpus callosum and pons), removal of cerebellum and brain stem, tessellation of WM boundary, smoothing of the tessellated surface and automated topology correction^62^. Using a deformable surface-based algorithm, the tessellated surface was used to find, first the WM boundary which was followed by the pial boundary. For every point on the tessellated WM surface, cortical thickness was calculated as an average representation of two distances: a) distance of a point on WM surface to the closest point on the pial surface and b) the distance from that point back to the closest point on the WM surface^62^. Cortical thickness were derived for 34 native-space gray-matter regions per hemisphere (total = 68 regions)^63^. These regions across all participants served as the feature set in the machine-learning (ML) framework, with targets as POB and SOB. The features and targets were used to derive a predictive model for the OBs, via a robust ML framework.

### Machine-learning framework

The ML framework was designed using scikit-learn (https://scikit-learn.org/stable/,^64^) via python implementation. We performed a nested cross-validation (CV) utilizing the feature and the target datasets. The nested CV approach ensures that the performance of the model is tested on the data that was not seen during model fitting, minimizing data leakage and overfitting. The entire framework was split into two CV schemes, i.e., the outer CV and the inner CV. The outer CV was used for the purpose of obtaining unbiased predictions of the target using the best model estimator, whereas the inner CV was performed to derive the best model estimator. The model that we used was LASSO (least absolute shrinkage and selection operator) regression, which is based on adding an L1-penality to the linear regression, for the purpose of a better generalization. Our main purpose of applying the LASSO model was for an easy interpretation of the results, as LASSO shrinks non-relevant features by assigning a zero weight. The best model estimator (inner CV) was selected by tuning the scaling factor α, which scales the L1-regularization. The values tested for α = [0.0001, 0.001, 0.01, 0.1, 10, 100, 1000], and the selection of the best α was based on mean squared error as the cost function. For both model selection (inner CV), as well as final predictions (outer CV), data was split using leave-one-out CV. All associations were tested for Pearson’s r, with p-value calculated based on 10, 000 surrogate combinations. In predictive modelling, it is often the case that the predictions are confounded/driven by variables that are of no prime interest to the research question, and can be detrimental to model relevance^26,65^. Hence, we also looked at potential correlations between the predictions and potential confounding measures, i.e., age, sex and TIV. In case the predictions were confounded by these measures, ultimately the confounded measures were linearly regressed out to make the predictions confounder-free^26^.

## Data Availability

The data will be available upon a reasonable request send to Dr. Tatjana Aue.
